# Fabrication of a Monolithic Computer-Aided Design (CAD)/Computer-Aided Manufacturing (CAM) Zirconia Crown Adapted to the Clasp of an Existing Removable Partial Denture

**DOI:** 10.7759/cureus.102028

**Published:** 2026-01-21

**Authors:** Jun Takebe

**Affiliations:** 1 Department of Removable Prosthodontics, Aichi Gakuin University, School of Dentistry, Nagoya, JPN

**Keywords:** cad/cam technology, dental education, existing clasp, gerodontology, provisional restoration, removable partial denture, zirconia crown

## Abstract

This case report illustrates a technique for fabricating a monolithic computer-aided design (CAD)/computer-aided manufacturing (CAM) zirconia crown by reproducing the morphology of a provisional restoration that had been adapted to the clasp of an existing removable partial denture. A 69-year-old patient presented after endodontic treatment of the mandibular right first and second premolars, which served as abutments for a removable partial denture with a double Akers clasp. To achieve optimal clasp adaptation, occlusal contacts, marginal fit, a rest seat, a guide plane, and undercut areas were intentionally incorporated into the provisional restoration. Models of the abutment teeth, provisional restoration, and opposing dentition were fabricated. A definitive cast was scanned with a digital scanner, and the datasets were superimposed to design and fabricate a monolithic zirconia crown. The crown demonstrated excellent clasp adaptation upon placement. Over a four-year follow-up period, denture movement was minimized, abutment teeth remained unaffected, and periodontal tissues remained stable. This case highlights the clinical effectiveness of using a provisional restoration incorporating clasp adaptation as a guide for the CAD/CAM-based fabrication of definitive monolithic zirconia crowns for abutment teeth.

## Introduction

In clinical practice, abutment teeth for removable partial dentures may require crown re‑treatment due to caries or endodontic therapy. When clasps on an existing removable partial denture can be replaced, it is common practice to fabricate new clasps after the abutment crowns are completed and then integrate them into the denture. However, when a removable partial denture with a rigid framework is in place, incorporating new clasps is difficult. In such cases, it is essential to fabricate a crown that properly fits the existing clasp. In the current era of a super-aged society, the long-term stability and functional maintenance of existing removable partial dentures have become increasingly important. Ensuring compatibility between abutment teeth and clasp assemblies is critical for maintaining denture stability and preventing functional deterioration. One conventional method involves the use of acrylic resin, particularly autopolymerizing resin [1-3]. First, an acrylic resin coping is created on the abutment teeth of the definitive cast. The coping is placed on the abutment teeth, the existing removable partial denture is connected, and acrylic resin [1-4] or a wax pattern [5] is built up while marking the inner surface of the clasp. The resin or wax pattern is then cast and polished to complete the crown. The fit of the abutment teeth, interproximal relationships with adjacent teeth, occlusal contacts, and compatibility with the existing clasp are subsequently evaluated. In recent years, advances in digital technology have enabled the precise recording of crown morphology. Moreover, computer-aided design (CAD)/computer-aided manufacturing (CAM) systems have facilitated the high‑precision fabrication of crowns, thereby enhancing the quality of prosthetic treatment for abutment teeth in removable partial dentures [6-8]. Provisional restorations play a critical role in prosthetic dental treatment. They preserve and restore the form and function of the stomatognathic system until the definitive crown is completed, and they also serve as essential guidelines for determining the design of the definitive crown prosthesis, including crown morphology, guide planes, and related structures [ 9]. Therefore, the author considers it feasible to reproduce the morphology and function of provisional restorations in definitive crowns by utilizing CAD/CAM technology. Furthermore, applying this technique makes it possible to fabricate crowns that properly adapt to the existing clasps of removable partial dentures. More recently, CAD/CAM technology has enabled the fabrication of definitive crown prostheses by replicating the morphology of the provisional restoration used during treatment, thereby expanding treatment options. In other words, if a provisional crown that fits the existing clasp can be obtained, the definitive crown can be fabricated using this copying technique without the need to remake the clasp. The author, therefore, proposes that this procedure may be beneficial in removable partial denture prosthetic treatment. In this case report, the author describes a single clinical example in which a provisional restoration adapted to an existing clasp was used as a digital blueprint for fabricating a definitive monolithic zirconia crown. The purpose of this report is to illustrate the clinical feasibility of this approach rather than to generalize its applicability. This technique may be useful in selected cases where modification of an existing removable partial denture is difficult.

## Case presentation

**Figure 1:**
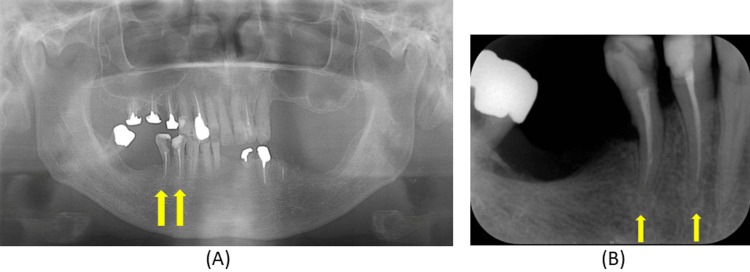
Radiographic findings at the initial examination (A) Panoramic radiographic findings; (B) Dental radiographic findings. These images provide baseline information on abutment tooth condition and help assess structural factors relevant to clasp engagement and long‑term prosthesis stability.

A 69‑year‑old patient presented with discomfort related to the mandibular right removable partial denture. The mandibular right first and second premolars, which served as abutments for a double Akers clasp, had previously undergone endodontic treatment (Figure 1).

**Figure 2:**
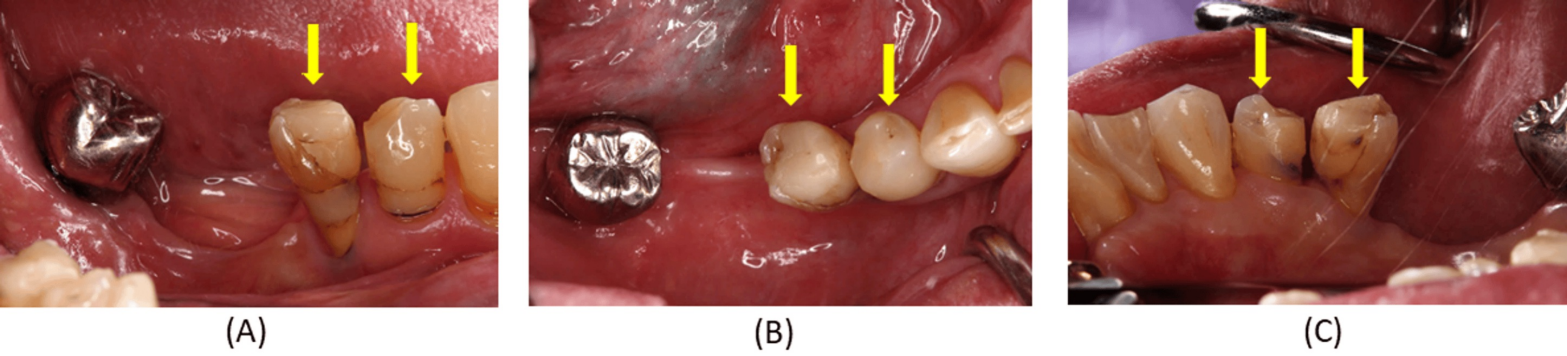
Intraoral findings without the denture at the initial examination. (A) Buccal view of the right mandibular region; (B) Occlusal view of the right mandibular region; (C) Lingual view of the right mandibular region. These views clarify the morphology of the abutment teeth and soft tissues before treatment, which is essential for planning clasp adaptation and crown contouring.

Carious lesions in these teeth were temporarily restored with composite resin (Figure 2).

**Figure 3:**
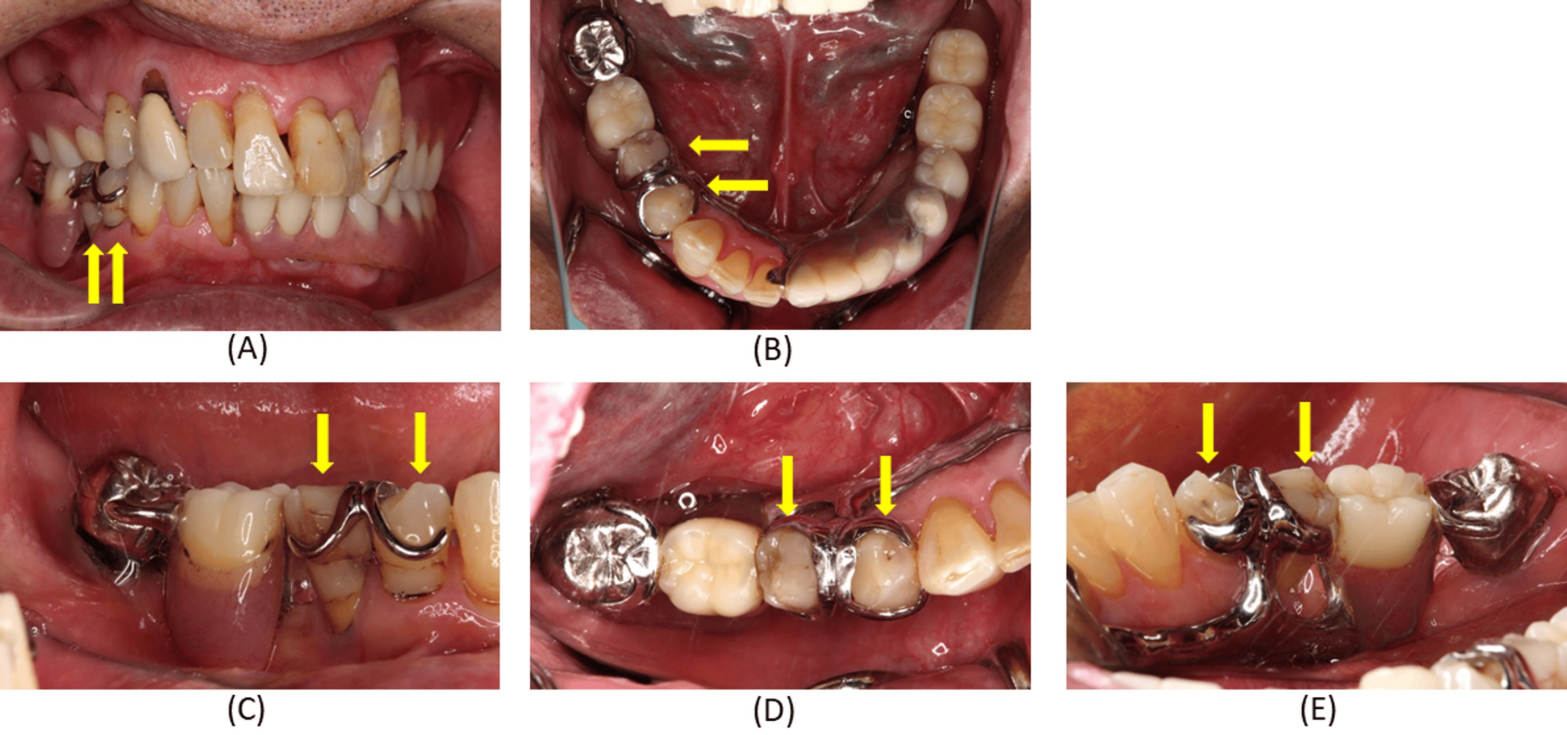
Intraoral findings with the denture in place at the initial examination. (A) Frontal view of the maxilla and mandible; (B) Occlusal view of the mandible; (C) Buccal view of the right mandibular region; (D) Occlusal view of the right mandibular region; (E) Lingual view of the right mandibular region. These images illustrate the relationship between the existing denture and the abutment teeth, highlighting areas where clasp contact and path of insertion must be preserved.

Although the existing removable partial denture was functional, clinical examination revealed insufficient clasp adaptation (Figure 3).

Periodontal tissues were healthy, and oral hygiene status was acceptable. The treatment objective was to fabricate definitive crowns for the mandibular right first and second premolars that would properly adapt to the existing clasp assembly. Provisional restorations were planned to evaluate clasp fit, occlusal contacts, marginal adaptation, rest seat form, guide plane configuration, and undercut areas.

**Figure 4:**
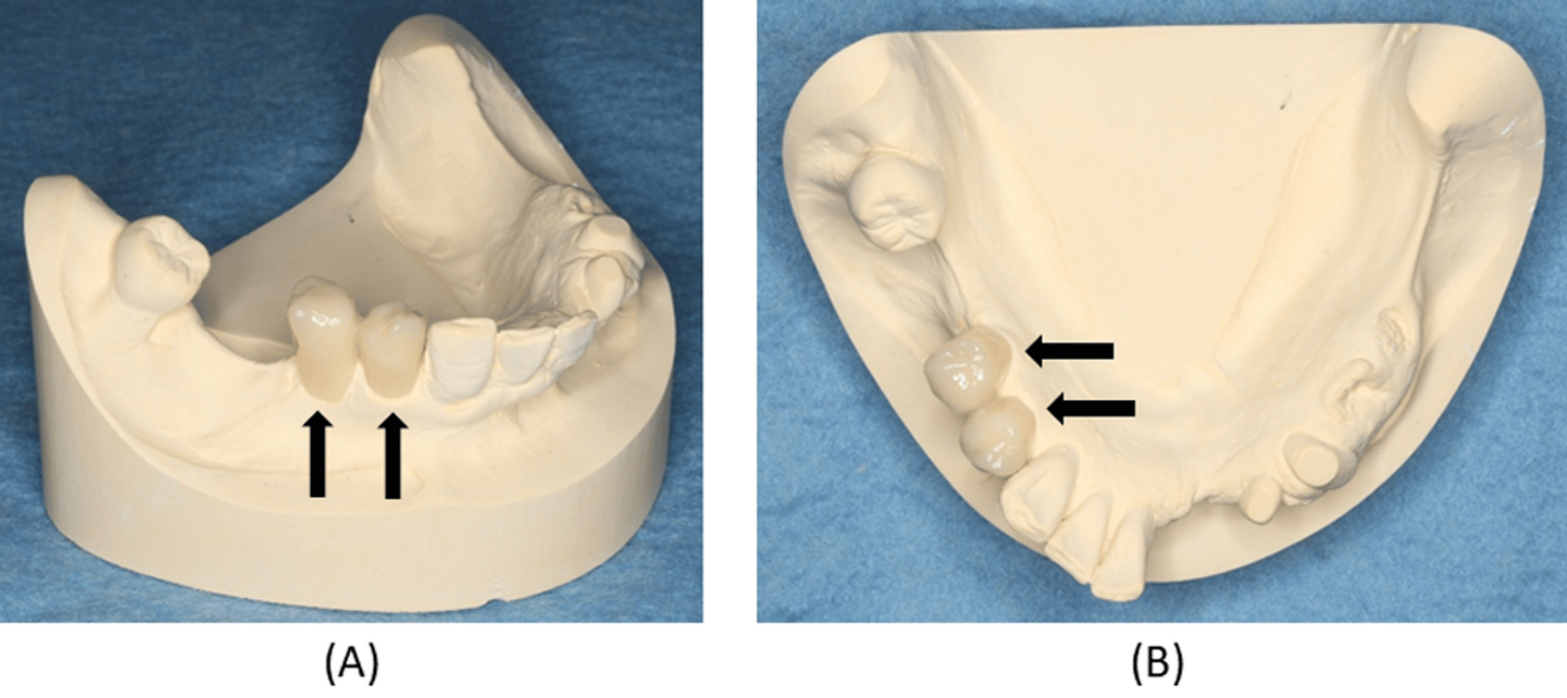
Provisional restoration for the right mandibular premolar region. (A) Occlusal view of the mandible; (B) Buccal view of the right mandibular region. The provisional restoration establishes the intended axial contours and clasp‑related surfaces that will be transferred to the definitive crowns.

A preliminary impression of the maxillary dentition was obtained with alginate impression material, followed by a silicone impression of the mandibular dentition. The maxillomandibular relationship was recorded in maximal intercuspal position with both dentures in situ. Casts were fabricated and digitized using a laboratory scanner, and provisional restorations were manufactured using CAD/CAM technology (Figure 4).

**Figure 5:**
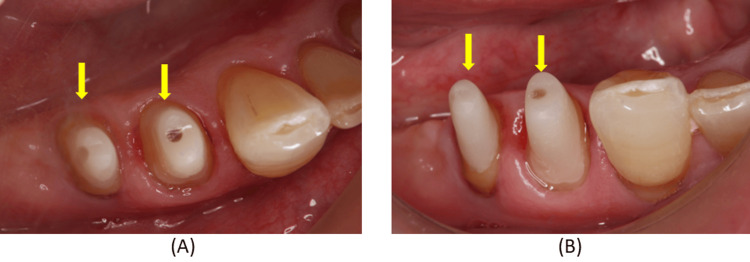
Intraoral findings of the prepared right mandibular first and second premolar abutments. (A) Occlusal view of the prepared right mandibular first and second premolar abutments; (B) Buccal view of the prepared right mandibular first and second premolar abutments. The prepared abutments show controlled reduction that preserves guide planes and undercut morphology necessary for clasp retention.

Foundation restorations were completed using a fiber‑reinforced composite resin post and composite resin cement. The post-cement complex was luted with resin cement. The foundation restorations were treated with the Rocatec system (CoJet™ Sand, 3M ESPE, Saint Paul, MN, USA), followed by application of a silane coupling agent (RelyX Ceramic Primer, 3M ESPE). After root canal preparation, the canal walls were conditioned with a 10‑3 solution of 10% citric acid and 3% ferric chloride for 30 seconds [10], and adhesive resin cement (RelyX™ Unicem 2 Automix Self‑Adhesive Resin Cement, 3M ESPE) was applied. Abutment tooth preparations for the mandibular right first and second premolars were then completed (Figure 5).

**Figure 6:**
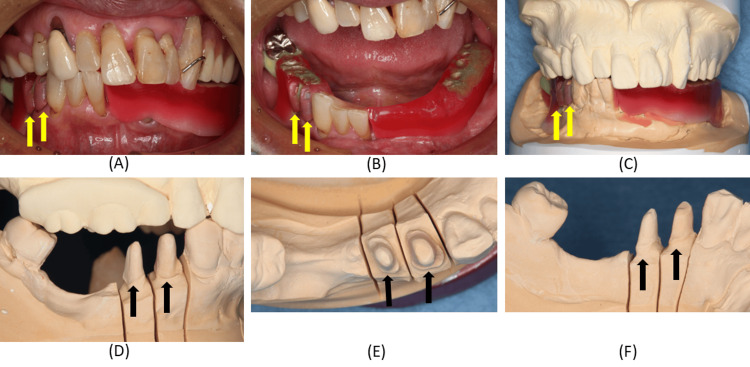
Intraoral findings during maxillomandibular relationship recording and findings of the right mandibular first and second premolar abutments after articulator mounting. (A) Frontal view during maxillomandibular relationship recording with the mandibular record base with occlusion rim in place; (B) Occlusal view of the mandible during maxillomandibular relationship recording with the mandibular record base with occlusion rim in place; (C) Frontal view of the maxillary and mandibular casts mounted on the articulator; (D) Buccal view of the right mandibular first and second premolar abutment teeth; (E) Occlusal view of the right mandibular first and second premolar abutment teeth; (F) Lingual view of the right mandibular first and second premolar abutment teeth. These steps ensure accurate transfer of occlusal relationships and abutment morphology, which is critical for fabricating crowns that integrate with the existing removable partial denture.

Precision impressions of the abutment teeth and opposing dentition were obtained, and a definitive cast was fabricated. A record base with an occlusion rim was constructed, and the maxillomandibular relationship was recorded (Figure 6).

**Figure 7:**
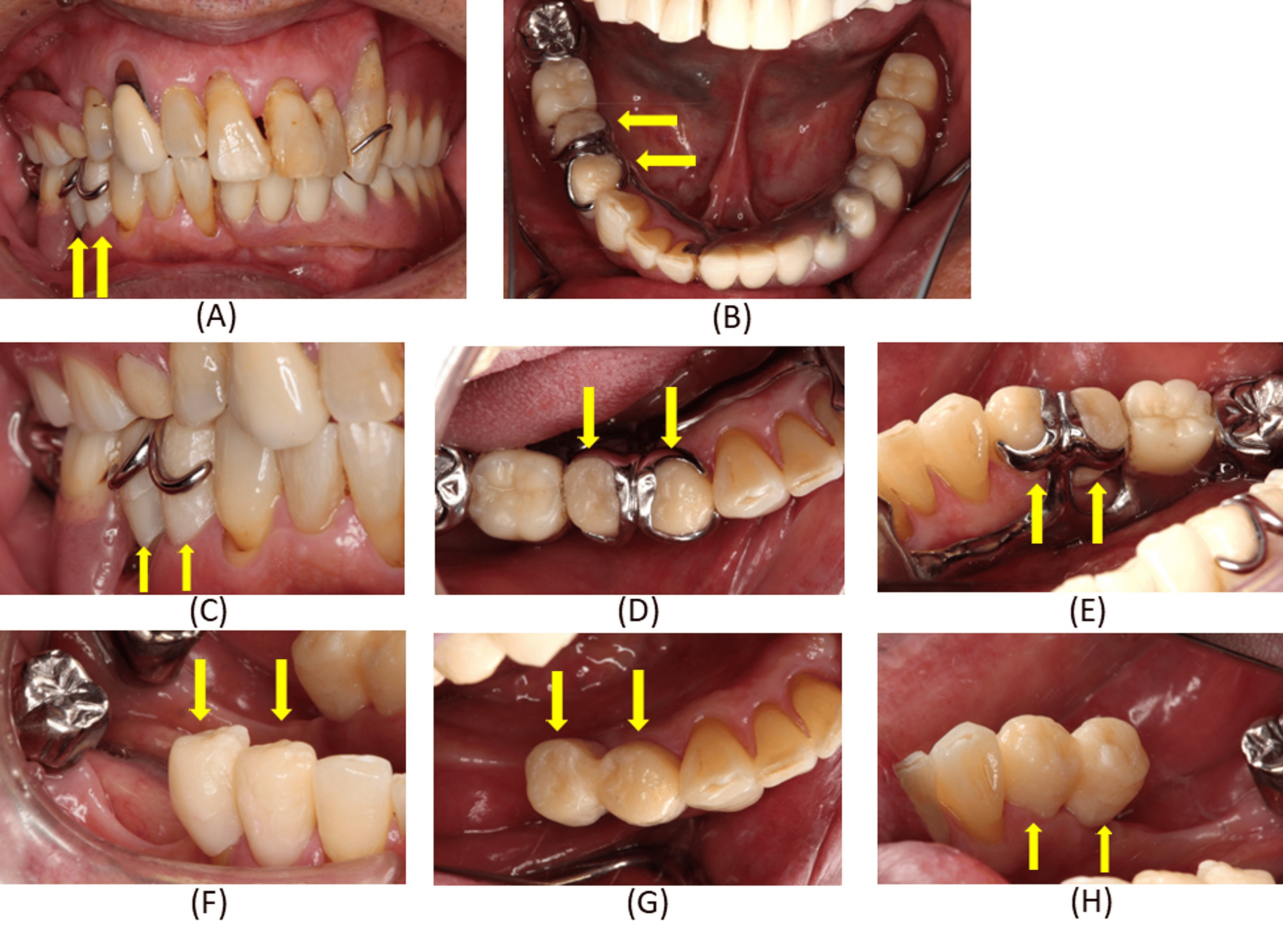
Intraoral findings after placement of the provisional restorations on the right mandibular first and second premolars. (A) Frontal view of the provisional restoration adapted to the clasp; (B) Mandibular occlusal view of the provisional restoration adapted to the clasp; (C, F) Buccal views of the provisional restoration adapted to the clasp with and without the denture in place; (D, G) Occlusal views of the provisional restoration adapted to the clasp with and without the denture in place; (E, H) Lingual views of the provisional restoration adapted to the clasp with and without the denture in place. These images confirm that the provisional restorations reproduce clasp‑related contours and maintain the original path of insertion of the denture.

The provisional restorations were tried in, and autopolymerizing resin was applied to refine the axial and occlusal surfaces. Adjustments were made to ensure proper rest seat form, bracing, and retentive clasp contact, guide plane adaptation, occlusal and proximal contacts, and cervical fit. After provisional cementation, it was confirmed that the patient could comfortably insert and remove the removable partial denture (Figure 7).

An impression of the fitted provisional restorations was then obtained using silicone impression material, and a cast was fabricated to serve as a reference for definitive crown design. An impression of the opposing maxillary dentition was also obtained.

**Figure 8:**
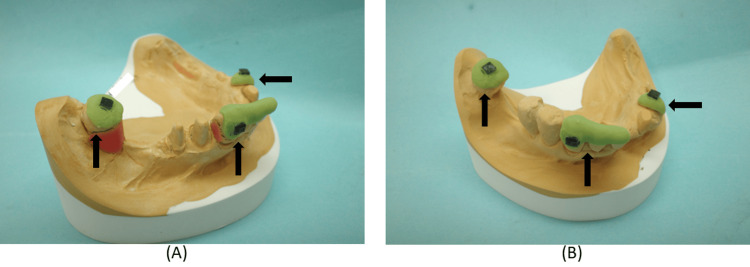
Occlusal landmarks added to the casts of the right mandibular first and second premolar abutment teeth (A) and their provisional restorations (B) for superimposition. Landmarks facilitate precise digital alignment, enabling accurate transfer of functional morphology to the definitive crowns.

To reproduce the clasp‑related morphology established with the provisional restorations, the datasets of the provisional restoration and the definitive cast were superimposed (DW‑7‑140, 7Series, Dental Wings Inc., Montréal, Quebec, Canada). Three anatomical landmarks on the models were used to align the datasets (Figure 8).

**Figure 9:**
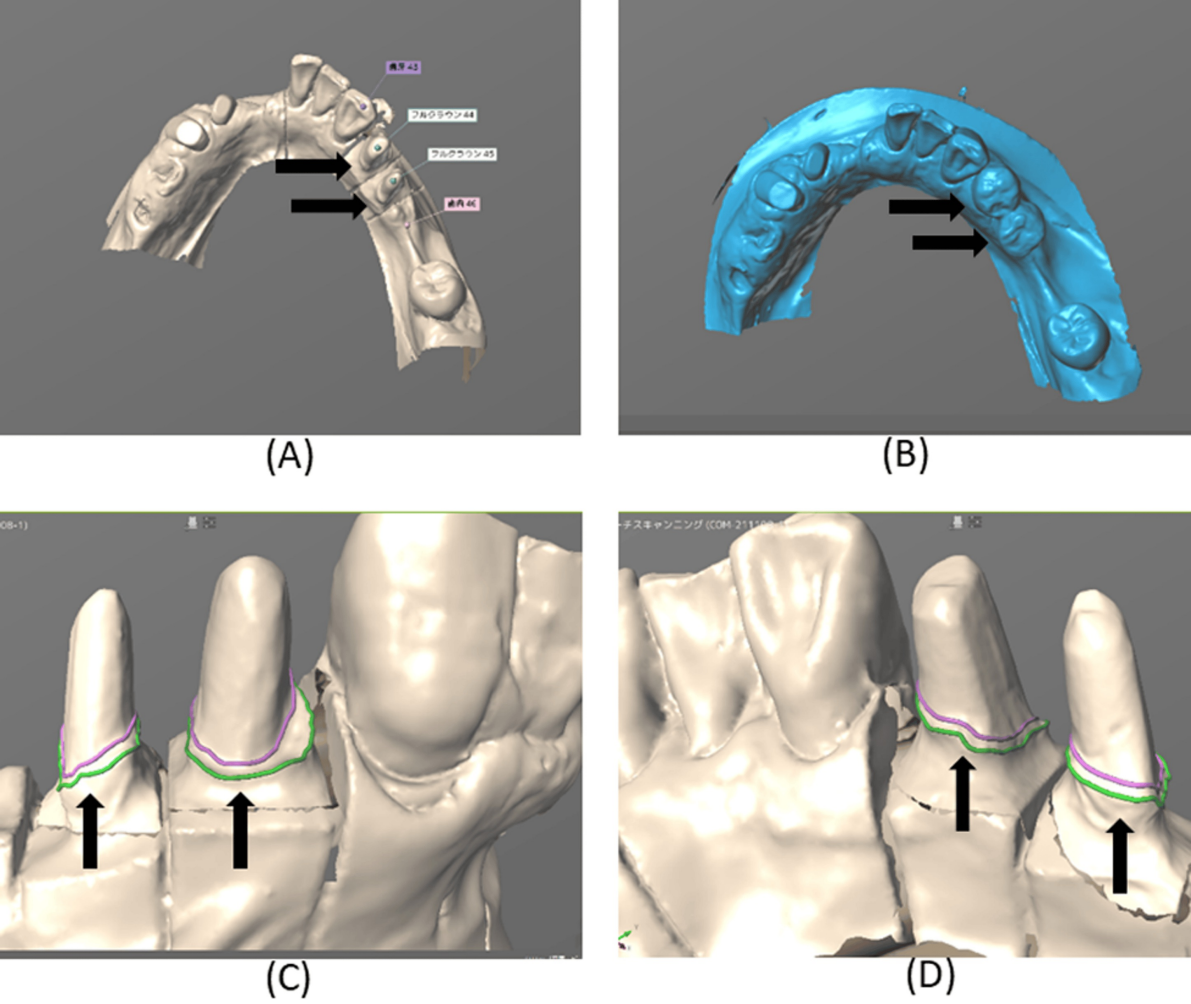
Digital scan data of the casts of the right mandibular first and second premolar abutment teeth (A) and their provisional restorations (B). (A) Occlusal view of the digitally scanned cast of the right mandibular first and second premolar abutment teeth; (B) Occlusal view of the digitally scanned cast of the right mandibular first and second premolar provisional restorations; (C) Buccal view of the digitally scanned cast of the right mandibular first and second premolar abutment teeth; (D) Lingual view of the digitally scanned cast of the right mandibular first and second premolar abutment teeth. Digital scanning captures fine morphological details, allowing the computer-aided design to replicate clasp‑related surfaces with high fidelity.

The CAD software (CARES Visual, Dental Wings Inc.) displayed both the abutment tooth model and the provisional crown model for verification (Figure 9).

**Figure 10:**
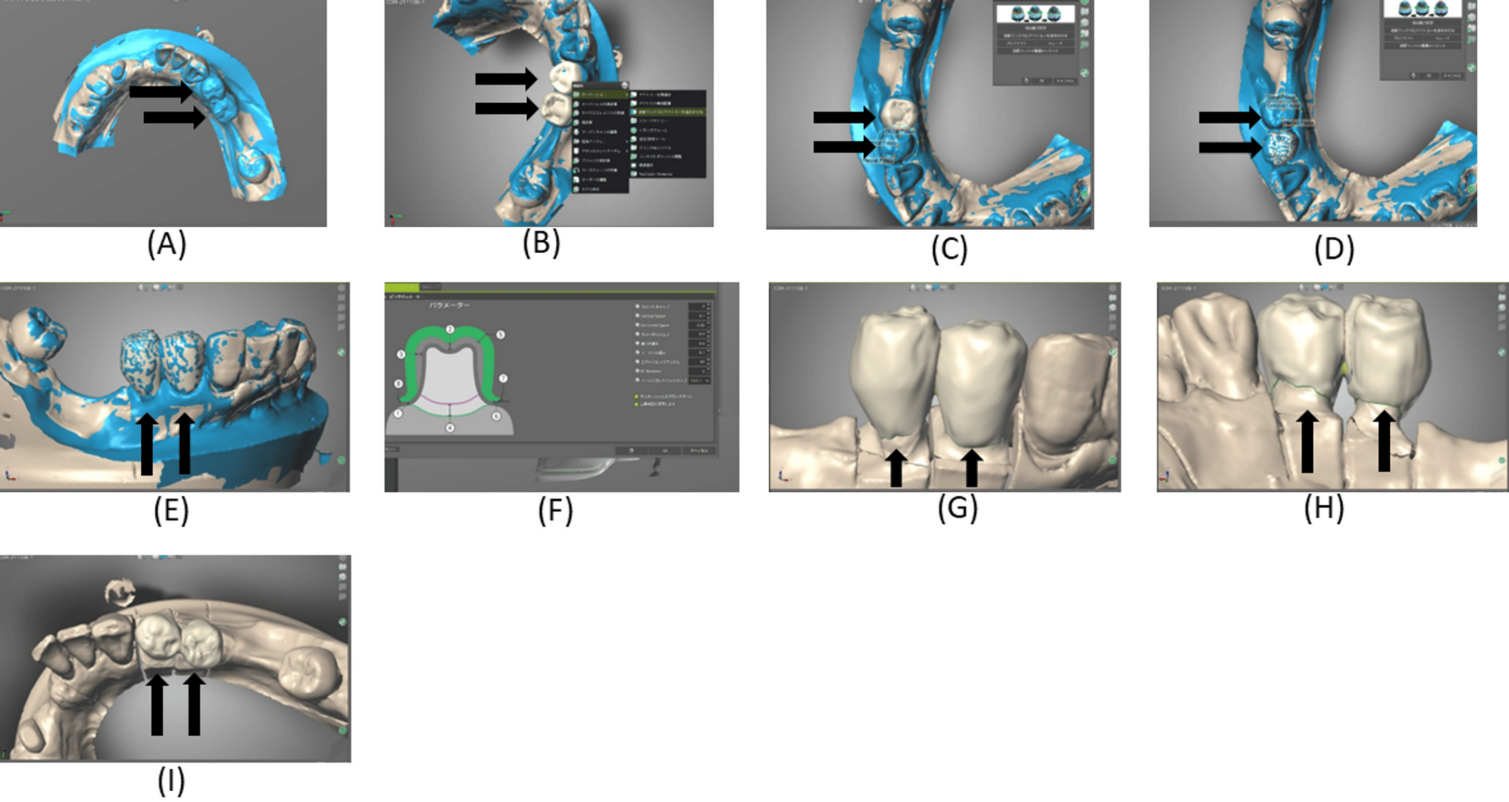
Design of the crown morphology on the digitally scanned cast of the right mandibular first and second premolar provisional restorations using the computer-aided design (CAD) software (A–E), followed by final crown morphology design with adjustment of the cement space (F–I). The CAD workflow enables controlled reproduction of axial contours while optimizing internal fit through cement space calibration.

The superimposed datasets were evaluated from multiple directions to confirm accurate alignment (Figures 10A-10E).

Cement space was set at 50-100 µm, as shown in the CAD design (Figures 10F-10I). Support structures were then added to complete the milling design.

**Figure 11:**
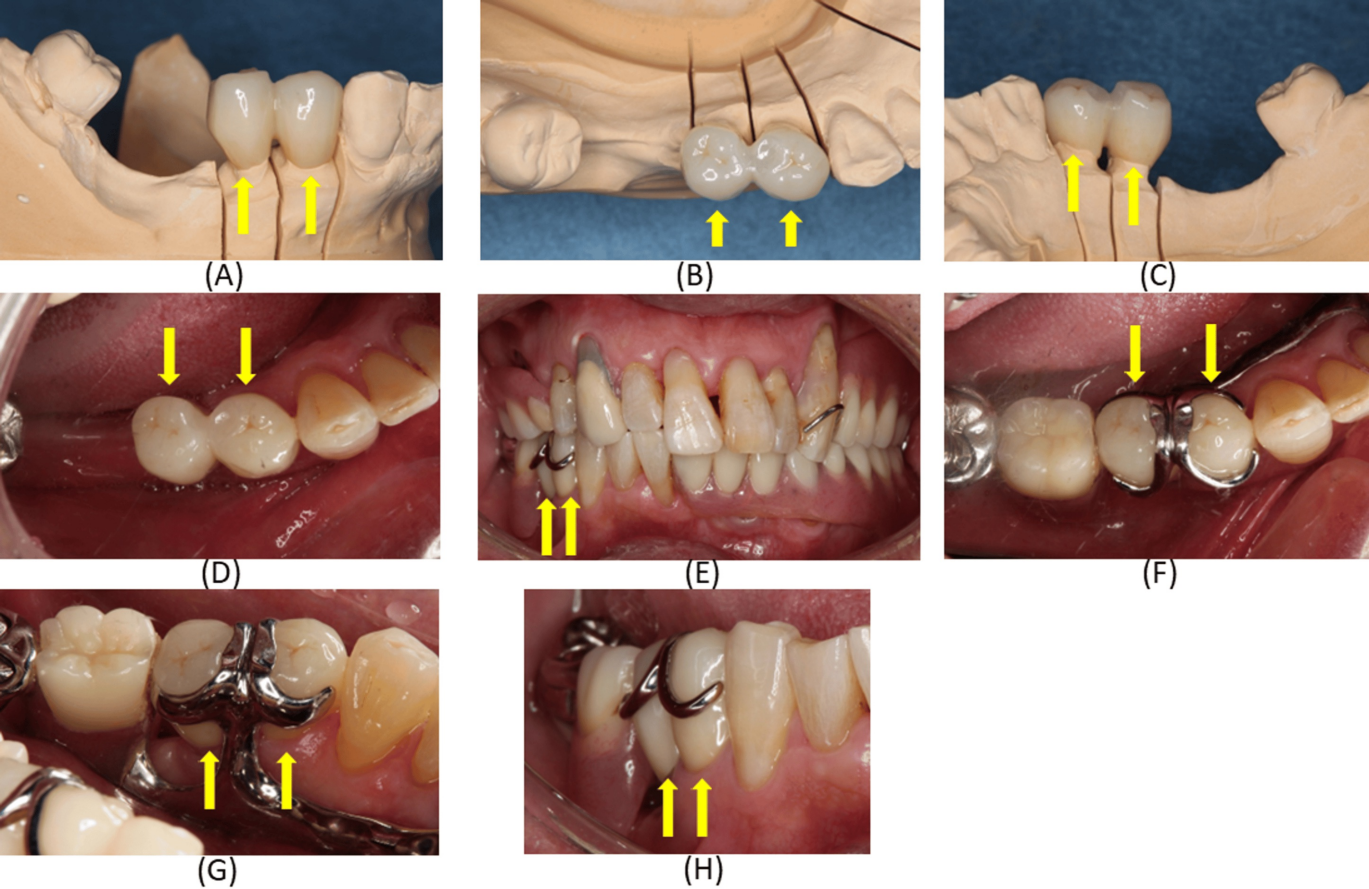
Completed monolithic computer-aided design/computer-aided manufacturing zirconia crowns and their placement on the right mandibular first and second premolars. (A-C) Completed crowns on the working model; (D) Occlusal view of the crowns cemented after try‑in and adjustment; (E) Frontal view of the crowns adapted to the clasp; (F) Mandibular occlusal view of the crowns adapted to the clasp; (G) Lingual views of the crown adapted to the clasp; (H) Buccal views of the crowns adapted to the clasp. These images demonstrate accurate reproduction of clasp‑contact areas and stable integration with the existing removable partial denture.

The crowns were milled (M series, Amann Girrbach, Mäder, Austria) from a monolithic zirconia disk (SHOFU Disk ZR Lucent Supra; Shofu Inc., Kyoto, Japan), sintered, and polished. Only minor adjustments were required, as the clasp‑related surfaces had been intentionally replicated from the provisional restorations (Figures 11A-11C).

At try‑in, crown margins, proximal contacts, occlusion, clasp fit, rest seat form, guide plane alignment, and undercut areas were evaluated. The inner surface of the clasp demonstrated intimate contact with the axial surface of the crown, indicating stable adaptation. The internal surface of the crowns was treated with tribochemical silica coating (Rocatec system, CoJet™ Sand, 3M ESPE) and subsequently coated with a silane coupling agent (RelyX Ceramic Primer, 3M ESPE). Prior to cementation, the abutment surfaces were conditioned with a 10‑3 solution of 10% citric acid and 3% ferric chloride following the protocol described by Taira and Imai [10]. The crowns were then luted using adhesive resin cement (RelyX™ Unicem 2 Automix Self‑Adhesive Resin Cement, 3M ESPE) (Figure 11D-H).

**Figure 12:**
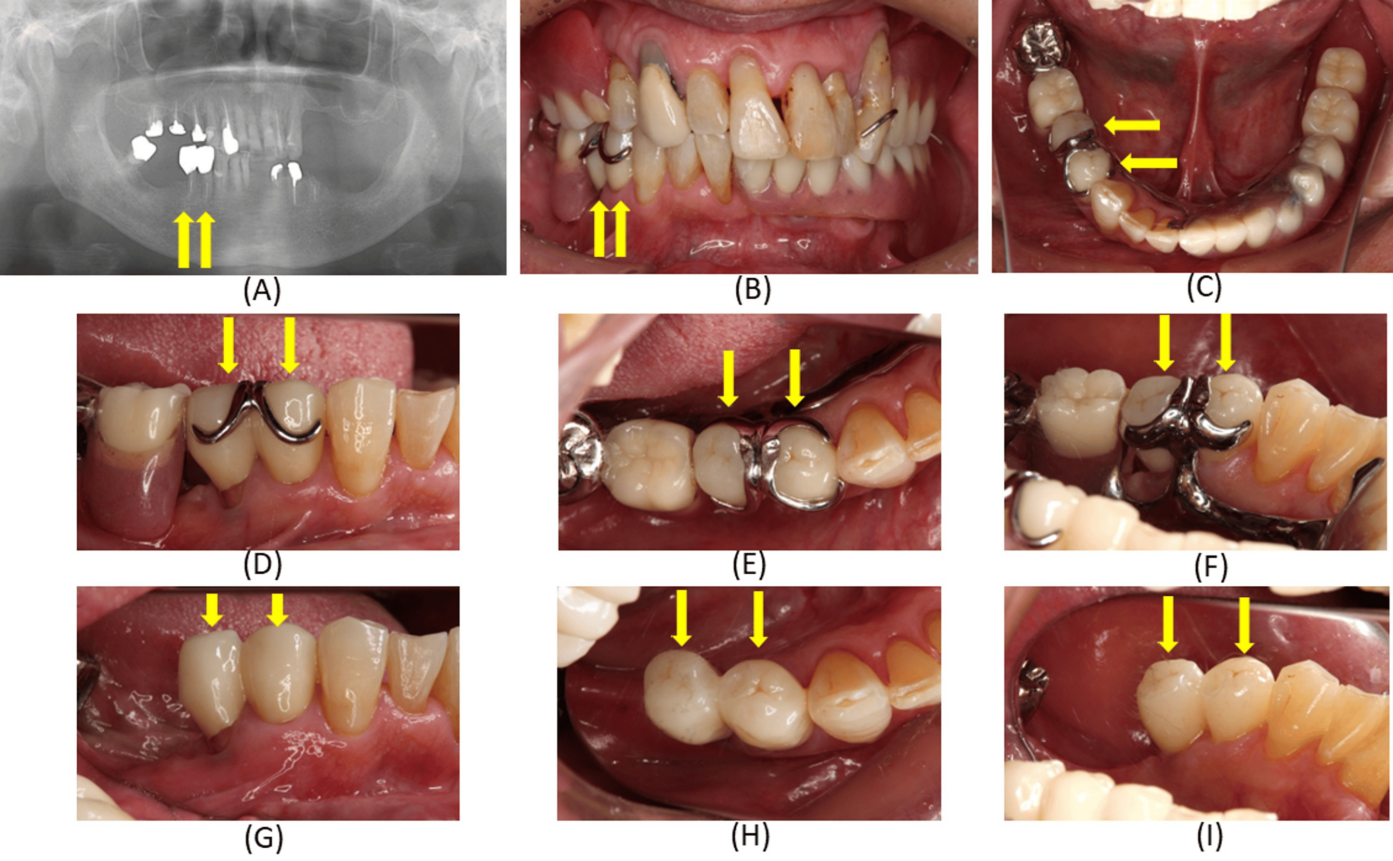
Radiographic and intraoral findings four years after placement of monolithic computer-aided design (CAD)/computer-aided manufacturing (CAM) zirconia crowns on the right mandibular first and second premolars. (A) Panoramic radiographic findings; (B-E) View of the crowns adapted to the clasp with the denture in place; (G-I) View of the crowns adapted to the clasp without the denture in place. The long‑term findings confirm stable clasp adaptation, healthy abutment teeth, and sustained functional performance of the prosthesis.

The patient was followed for four years. During this period, the zirconia crowns maintained stable clasp adaptation and functional integration with the removable partial denture. Denture movement was minimized, the abutment teeth remained healthy, and no periodontal complications were observed. Periodontal examinations revealed no abnormalities, with probing depths of 1-3 mm, no tooth mobility, and no bleeding on probing of the remaining maxillary and mandibular teeth or the residual mucosa. These findings remained unchanged and stable compared with the initial visit. At each follow‑up appointment, adherence to the intervention and tolerability regarding denture comfort were verbally assessed. The occlusion of the denture and the conformity of the basal surface were routinely examined to detect any abnormalities. Oral hygiene evaluations and maintenance were performed, together with assessments of masticatory function, to ensure that the patient was able to eat without difficulty. Following treatment, the patient demonstrated stable motivation for oral hygiene and an improved understanding of denture use, which contributed positively to the long‑term prognosis and overall quality of life. No problems with masticatory function were identified in a masticatory ability test using gummy jelly [11] or in interviews with the patient. Occlusal examination using articulating paper and occlusal registration strips confirmed a stable maxillomandibular relationship, with satisfactory comfort and masticatory function maintained throughout the follow‑up period (Figure 12).

## Discussion

In removable partial dentures, rigid support is considered advantageous, and metal‑base dentures with highly rigid frameworks are regarded as effective prostheses [11]. When prosthetic crown treatment is required for an abutment tooth, particularly in cases where the removable partial denture includes a metal framework or where clasp removal is difficult, fabricating a crown with a contour that accommodates the existing clasp allows the patient to continue using their current denture. This approach preserves the functional stability of the prosthesis and maintains patient comfort and familiarity with the appliance.

Various techniques for fabricating crowns that fit existing clasps have been reported. In the conventional method, a resin coping made of autopolymerizing resin is fabricated on the working model, followed by waxing and intraoral try‑in. The existing clasp is softened in hot water and tried in the mouth, after which the undercut and guide plane are adjusted and added [5]. Another method involves fabricating a resin coping on the working model, performing an intraoral try‑in, and then applying additional autopolymerizing resin to adapt the coping to the clasp. The coping is returned to the working model, and wax is used to complete the surveyed crown morphology [1-4].

However, heat generated during waxing may deform the resin coping or cause casting defects [12], making the quality of the prosthesis highly dependent on the dental technician’s expertise. To address these limitations, alternative approaches using CAD/CAM technology have been introduced [6-8, 13-17]. When monolithic zirconia is selected as the definitive material, casting is not feasible, further underscoring the importance of CAD/CAM systems in contemporary crown fabrication.

The application of CAD/CAM technology enables the fabrication of crowns that fit existing clasps with greater precision, eliminating the complex and time‑consuming laboratory procedures required in earlier methods [16, 17]. The documented accuracy of CAD/CAM systems supports their use in situations where the morphology of a provisional restoration must be transferred precisely to the definitive crown [13]. Techniques have been reported for reproducing planned crown morphology by scanning a pre‑treatment diagnostic model of the tooth targeted for prosthetic treatment [8, 15]. This approach is effective when a pre‑treatment model is available.

In the present case, the mandibular right first and second premolars serving as abutment teeth had undergone endodontic treatment, resulting in loss of tooth structure. Provisional restorations adapted to the existing clasps were fabricated to restore the original crown morphology. The provisional state was then scanned, enabling reproduction of the crown form for the definitive prosthesis. A precision impression of the provisional crown morphology was obtained to fabricate a definitive cast. After the definitive cast was completed, both casts were scanned and superimposed using CAD/CAM technology to design the definitive crown, and a monolithic zirconia-surveyed crown was fabricated.

Monolithic zirconia has been reported as a suitable material for crowns serving as abutments for removable partial dentures [18, 19]. The improved strength and durability of current zirconia materials provide a strong rationale for their selection in cases requiring stable clasp adaptation [14]. Clinical studies have shown that monolithic zirconia is an excellent alternative to metal-ceramic crowns, with surveyed monolithic zirconia crowns demonstrating high survival rates after seven years of service [20]. In the present case, four years have elapsed since crown placement, and no clinical complications have been observed, indicating a favorable outcome.

This case study has several limitations. First, the axial surfaces of the provisional restorations, along which the clasp runs, required multiple adjustments using autopolymerizing resin to achieve optimal adaptation. Although a satisfactory fit was ultimately obtained, repeated modifications were necessary. Provisional restorations with a well‑defined axial contour could potentially be fabricated by recording the clasp path intraorally using a direct resin technique, followed by optical impression taking with an intraoral scanner. Second, in this case, the adjusted provisional restorations were temporarily attached to the abutment teeth, after which a precision silicone impression was obtained, and a superhard plaster model was fabricated. This model was then scanned to produce the monolithic zirconia crown. Direct scanning and duplication of the adjusted provisional restorations would likely facilitate fabrication; however, this was not possible because the necessary CAD/CAM equipment was not available at the author’s institution.

Provisional restorations play a critical role in the success of crown prosthetic treatment and are of considerable clinical significance [9]. To the best of our knowledge, to date, no reports have described fabricating a definitive crown by directly utilizing a provisional restoration that fits an existing clasp, as performed in this case. Allowing the patient to wear the provisional crown in daily life enables confirmation of the appropriateness of the crown morphology intended for the final prosthesis. Although some adjustments were required to refine the provisional restoration, this approach proved highly effective. The crown morphology was validated in its function as an abutment tooth, and the contour of the provisional restoration was directly transferred to the monolithic CAD/CAM zirconia crown. This process ensured functional adaptation and continuity with the patient’s existing removable partial denture, thereby enhancing the overall effectiveness of the prosthetic treatment. In an increasingly super-aged society, maintaining the stability and functionality of existing dentures through continuous maintenance is essential for preserving the quality of life and daily functioning of older adults [11]. Future studies involving a larger number of cases are warranted to further validate the clinical applicability of this approach.

## Conclusions

This study demonstrated that it is feasible to fabricate a crown that precisely conforms to the clasp path of an existing removable partial denture. When prosthetic crown treatment is required for an abutment tooth, removal of the clasp may be challenging. In such situations, applying the design guidelines for the definitive prosthesis becomes clinically effective, underscoring the important role of provisional restorations. This case further highlighted the usefulness of employing provisional restorations with clasp adaptation in the design and fabrication of a definitive crown using monolithic CAD/CAM zirconia technology. This approach ensured continuity with the existing denture, improved treatment efficiency, and emphasized the clinical value of provisional restorations in guiding the design of definitive prostheses. Clinically, this method reinforces the role of provisional restorations not only as provisional prostheses but also as diagnostic and design tools that directly influence the accuracy, functionality, and long‑term success of definitive prosthetic treatment. By integrating clasp adaptation into the provisional stage, clinicians can achieve predictable outcomes, minimize patient discomfort, and preserve the integrity of existing removable partial dentures.
